# Prevalence and treatment of diabetes mellitus and hypertension among older adults with intellectual disability in comparison with the general population

**DOI:** 10.1186/s12877-017-0658-2

**Published:** 2017-11-23

**Authors:** Anna Axmon, Gerd Ahlström, Peter Höglund

**Affiliations:** 1 Division of Occupational and Environmental Medicine, Faculty of Medicine, Lund University, SE-221 00 Lund, Sweden; 2 Department of Health Sciences, Faculty of Medicine, Lund University, Lund, Sweden; 3 Division of Clinical Chemistry and Pharmacology, Faculty of Medicine, Lund University, Lund, Sweden

**Keywords:** Aged, Cardiovascular disease risk factors, Drug prescription, Middle-aged

## Abstract

**Background:**

Diabetes mellitus and hypertension are risk factors for cardiovascular disease, which is the most common cause of death in the world. People with intellectual disability (ID) have been reported to have high rates of both these disorders. The aim of this study was to describe and compare prevalence ratios of diabetes mellitus and hypertension between older adults with ID and their age peers in the general population, and to describe and compare treatment patterns in these two groups.

**Methods:**

This is a Swedish register-based study, in which we established a cohort of people aged 55+ years and who had received support for those with ID in 2012 (*n* = 7936). We also established a same-sized referent cohort from the general population matched by sex and year of birth. Information on diagnoses of diabetes mellitus and hypertension, and prescription of drugs for these disorders, were collected from national registers for the period 2006–2012. The two cohorts were compared using generalized linear models (GLM).

**Results:**

People with ID were 20% more likely than the general population to have a diagnosis of diabetes mellitus, and 26% more likely to have prescription of drugs for diabetes mellitus. People in the general population were 81% more likely to have a diagnosis of hypertension, and 9% more likely to have a prescription of drugs for hypertension. Among those with diabetes, ID was associated with higher occurrence of prescription of insulin combination drugs and sulfonylureas, but lower occurrence of prescription of dipeptidyl peptidase (DPP) 4-inhibitors and exenatide/liraglutide. Among those with hypertension, ID was associated with higher occurrence of prescription of diuretics, but lower occurrence of prescription of calcium channel blockers and angiotensin II antagonists.

**Conclusions:**

Treatment regimens among people with ID tended to include older types of medication compared with what was prescribed in the general population. To ensure that this is medically appropriate and not due to failure to update the treatment regimen, it is important to investigate if the people with ID and diabetes mellitus or hypertension are subjected to the same regular drug reviews that are recommended for older adults in general.

**Electronic supplementary material:**

The online version of this article (10.1186/s12877-017-0658-2) contains supplementary material, which is available to authorized users.

## Background

Cardiovascular disease (CVD), such as myocardial infarction and stroke, is the leading noncommunicable disease as well as the most common cause of death in the world [1]. Alongside several lifestyle factors, hypertension and diabetes mellitus have been found to be risk factors for CVD. Thus, reductions of these disorders should reasonably lead to reductions in CVD and related deaths.

In the general population [2–4] as well as among people with intellectual disability (ID) [5–9], increasing age is a risk factor for both hypertension and diabetes mellitus. Adults with ID seem to experience higher rates of diabetes mellitus [10–13] than people without ID. If this effect is found also in older age groups, i.e. if the age effect is similar in people with and without ID, has been less studied. The few results published so far suggest that the increase in diabetes mellitus risk among people with ID compared to the general population decrease with age [12], which translates into a smaller age effect among people with ID. With respect to hypertension and ID, existing literature points towards similar rates among people with ID and in the general population [7, 8, 14], although this may be an effect of decreased rates among men and increased rates among women [7]. Nevertheless, studies focusing on older adults with ID are scarce and as they may constitute a group particularly burdened by diseases that are risk factors for CVD, further knowledge is needed in this area.

There is a variety of pharmacological treatments for diabetes mellitus, each associated with its own potential side effects [15]. Decision on treatment regime is facilitated by a range of guidelines [16–19]. However, in older patient groups, the guidelines for younger populations may not be relevant. For example, the use of metformin – which is the primary choice of drug in younger people – is often limited for older people because of comorbid conditions such as chronic renal insufficiency and heart failure [20]. To the best of our knowledge, treatment use among older people with ID and diabetes mellitus has not yet been described in scientific literature.

Unlike for diabetes mellitus, hypertension can be treated with the same drugs in older people as in younger age groups [21, 22]. Based on a high prevalence, van de Louw et al. [8] called for people with ID and hypertension to be treated in the same manner as in the general population, following national guidelines. However, as far as we know, there are no data published regarding actual treatments used among older people with ID and hypertension. Indeed, only a few years ago, de Winter et al. [7] concluded that a policy on prevention, detection and treatment of risk factors for CVD among people with ID was urgently needed.

In order to get health policies in place for older people with ID – a vulnerable group in which many have a lifelong experience of difficulties in expressing their illnesses and health care needs – increased knowledge is essential. Not only knowledge concerning the prevalence of disorders and their treatment per se, but also in comparison with age peers in the general population. Such information could play a vital role in determining which types of prophylactic health practices should be used to delay the onset and minimize the occurrence of life threatening CVD. Therefore, the aim of this study was to describe and compare prevalence ratios of diabetes mellitus and hypertension between older adults with ID and their age peers in the general population, and to describe and compare treatment patterns in these two groups. Moreover, to investigate differences in prevalence ratios stratified by sex, i.e. separately for men and women, as well as assess possible sex differences within each cohort.

## Methods

This is a register based study, using Swedish national registers both to define the original cohorts, identify the subcohorts with diagnosis of diabetes mellitus or hypertension, and collect information on outcomes (drug prescriptions).

### Registers

The **LSS register** contains information on all support and service provided according to the Act Concerning Support and Service for Persons with Certain Functional Impairments [23] (Swedish abbreviation: LSS). According to this act, a person with ID or autism spectrum disorder (ASD) may apply to his/her municipality for the support needed to optimize his/her opportunity to achieve good living conditions in his/her daily life. The act lists ten measures of support, of which eight are provided for adults. These include e.g. daytime activities, personal assistant, and special housing. The latter is in the form of private apartments with access to common areas. For those with less need of assistance, the common areas are normally in the proximity to the apartments, and staff is accessible around the clock, whereas for those with higher need of assistance, the common areas are adjoining the apartments and staff is on site at all times.

Although a diagnosis of either ID or ASD is required to receive the support, the diagnosis itself is not recorded in the register.

The **National Patient Register** contains records of all inpatient and outpatient specialist visits in Sweden. Inpatient visits are recorded at the date of discharge. For each visit, one primary and up to 21 secondary diagnoses are recorded, coded according to the International Statistical Classification of Diseases and Related Health Problems 10th Revision (ICD-10).

The **Prescribed Drug Register** contains information on all purchases of prescribed drugs in Sweden, corresponding to 84% of the total volume of drugs, starting July 2005 [24]. Purchases are registered at the date of dispensation, and information on the drug is given according to the Anatomical Therapeutic Chemical (ATC) classification system.

### Study cohorts

Information about the two original cohorts has been given in detail elsewhere [25–27]. Briefly, an administrative cohort of people with ID (*n* = 7936) was established using the LSS register. Moreover, a referent cohort was randomly selected from the general Swedish population by Statistics Sweden, one-to-one matched on sex and year of birth (gPop). All people in both cohorts were 55+ years old and alive at the end of 2012. For the ID cohort, information on residence in special housing, as described above, was collected from the LSS register, and was categorized as having lived in special housing at least one year or never during the study period.

### Diagnoses

Data regarding all diagnoses (primary as well as secondary) in inpatient care and at outpatient specialist visits during 2006–2012 were collected from the Swedish National Patient Register. Diagnostic codes E10 (insulin-dependent diabetes mellitus, or type 1 diabetes), E11 (non-insulin-dependent diabetes mellitus, or type 2 diabetes), E12 (malnutrition-related diabetes mellitus), E13 (other specified diabetes mellitus), and E14 (unspecified diabetes mellitus) were considered as diabetes mellitus, and I10 (essential [primary] hypertension), I11 (hypertensive heart disease), I12 (hypertensive renal disease), I13 (hypertensive heart and renal disease), and I15 (secondary hypertension) were considered as hypertension. All diagnoses made during the study period are listed in Additional file 1.

### Drug prescriptions

Information on purchases of prescribed drugs was collected for the period 2006–2012 from the Swedish Prescribed Drug Register. Drugs investigated as drugs for diabetes mellitus were those with ATC code A10. These were aggregated into third (e.g. A10B, blood glucose lowering drugs excluding insulins) and fourth (e.g. A10BA, biguanides) ATC level groups. As drugs for hypertension we considered those with ATC codes C03 (diuretics), C07 (beta blocking agents), C08 (calcium channel blockers), and C09 (agents acting on the renin-angiotensin system). These were analyzed separately, and also aggregated to ACE (Angiotensin-Converting-Enzyme) inhibitors (C09A & C09B) and angiotensin II antagonists (C09C & C09D). All drugs prescribed during the study period are listed in Additional file 2.

### Statistics

Analyses of dichotomous outcomes (e.g. having at least one prescription) were performed comparing people with ID to the gPop sample using generalized linear models (GLM) with a Poisson distribution, log link function and robust covariance matrix estimator, thus estimating relative risks (RRs) with 95% confidence intervals (CIs). Secondary analyses were performed comparing people with ID to the gPop sample stratified by sex. Moreover, we investigated sex differences in drug prescription within each cohort. Statistical interaction was assessed by introducing the cross product of sex and cohort affiliation in separate models.

All analyses regarding diabetes mellitus were stratified by type of diabetes mellitus, including only those with at least one diagnosis of either insulin- or non-insulin-dependent diabetes mellitus. Thus, 44 people with diagnosis of unspecified diabetes mellitus only, and one person with diagnosis of other specified diabetes mellitus were excluded.

All analyses were performed in SPSS version 23.0. *P*-values below 0.05 were considered statistically significant. Analyses were only performed when both groups to be compared contained at least five people.

## Results

### Disease prevalence

As both diabetes mellitus and hypertension are chronic disorders, the prevalence at the end of the study can be estimated by using the number of diagnoses and prescriptions during the study period.

For all proxies of diabetes mellitus (combinations of prescription and diagnosis), the people in the ID cohort had a higher prevalence compared with the gPop cohort (Table 1). This was driven by a higher prevalence among women, as no difference in occurrence of diabetes mellitus was found among the men. Within the gPop cohort, a higher risk of diabetes mellitus diagnosis was found for men (RR 1.64 [1.39–1.95]). The same effect was not found in the ID cohort (0.93 [0.81–1.08]).

**Table 1:** Prevalence estimates of diabetes mellitus and hypertension based on diagnosis and prescription used to treat each respective disorder in a cohort of older people with intellectual disability (ID) and a referent sample in the general population (gPop)

	All (*n* = 7936 in each cohort)			Women (*n* = 3609 in each cohort)			Men (*n* = 4327 in each cohort)			
	gPop	ID	ID vs gPop	gPop	ID	ID vs gPop	gPop	ID	ID vs gPop	
	*n* (%)	*n* (%)	RR (95% CI)	*n* (%)	*n* (%)	RR (95% CI)	*n* (%)	*n* (%)	RR (95% CI)	*p*
Diabetes mellitus										
Diagnosis (all)	616 (8)	741 (9)	**1.20** (**1.08–1.34**)	207 (6)	350 (10)	**1.69** (**1.42–2.01**)	409 (9)	391 (9)	0.96 (0.83**–**1.10)	**<0.001**
Diagnosis (insulin-dependent)	174 (2)	182 (2)	1.05 (0.85–1.29)	67 (2)	78 (2)	1.16 (0.84–1.61)	107 (2)	104 (2)	0.97 (0.74–1.27)	0.40
Diagnosis (non-insulin-dependent)	546 (7)	664 (8)	**1.22** (**1.09–1.36**)	186 (5)	311 (9)	**1.67** (**1.39–2.01**)	360 (8)	353 (8)	0.98 (0.85**–**1.14)	**<0.001**
Prescription	805 (10)	1013 (13)	**1.26** (**1.15–1.38**)	273 (8)	487 (13)	**1.78** (**1.54–2.07**)	532 (12)	526 (12)	0.99 (0.88**–**1.12)	**<0.001**
Diagnosis and prescription	541 (7)	671 (8)	**1.24 (1.11–1.29)**	179 (5)	314 (9)	**1.75 (1.46–2.11)**	362 (8)	357 (8)	0.99 (0.85–1.14)	**<0.001**
Diagnosis and/or prescription	880 (11)	1083 (14)	**1.23** (**1.13–1.35**)	301 (8)	523 (14)	**1.74** (**1.51–2.00**)	579 (13)	560 (13)	0.97 (0.86**–**1.09)	**<0.001**
Hypertension										
Diagnosis	1487 (19)	814 (10)	**0.55** (**0.50–0.60**)	629 (17)	358 (10)	**0.57** (**0.50–0.65**)	858 (20)	456 (11)	**0.53** (**0.47–0.60**)	0.44
Prescription	3812 (48)	3501 (44)	**0.92** (**0.88–0.96**)	1715 (48)	1689 (47)	0.99 (0.92–1.05)	2097 (48)	1812 (42)	**0.86** (**0.81–0.92**)	**0.005**
Diagnosis and prescription	1457 (19)	795 (10)	**0.55 (0.50–0.60)**	516 (17)	348 (10)	**0.57 (0.50–0.64)**	841 (19)	447 (10)	**0.53 (0.47–0.60)**	0.49
Diagnosis and/or prescription	3842 (48)	3520 (44)	**0.92** (**0.88–0.96**)	1728 (48)	1699 (47)	0.98 (0.92–1.05)	2114 (49)	1821 (42)	**0.86** (**0.81–0.92**)	**0.005**

Statistically significant results are marked in bold

*RR* relative risk, *CI* confidence interval, *ID* intellectual disability, *gPop* general population, *p* p-value for interaction between sex and cohort

A higher prevalence for the ID cohort was also found for non-insulin-dependent diabetes mellitus, but not for insulin-dependent diabetes mellitus (Table 1). Within the gPop cohort, men had a higher risk than women of non-insulin-dependent (RR 1.61 [1.35–1.93]) as well as insulin-dependent diabetes mellitus (1.33 [0.98–1.81]), although statistical significance was not achieved for the latter. No statistically significant differences were found between sexes in the ID cohort, neither for non-insulin-dependent (men vs women 0.95 [0.81–1.10]) nor insulin-dependent diabetes mellitus (1.11 [0.83–1.29]). Special housing was associated with a lower risk of both non-insulin-dependent (0.70 [0.59–0.83]) and insulin-dependent diabetes mellitus (0.80 [0.57–1.11]), although the latter was not statistically significant.

People with ID were less likely to have hypertension than the people in the gPop sample (Table 1). This was true for all proxies (combinations of prescription and diagnosis) of hypertension when examining the whole cohorts. However, when stratifying by sex, proxies including prescription of hypertension drugs was not associated with a higher prevalence for women with ID compared to women in the gPop cohort. Within the gPop cohort, men were statistically significantly more likely than women to have a diagnosis of hypertension (RR 1.14 [1.03–1.26]). The corresponding sex difference was not found in the ID cohort (men vs women 1.06 [0.93–1.22]). However, within the ID cohort, special housing was associated with a lower risk of being diagnosed with hypertension (0.70 [0.60–0.82]).

### Prescription of drugs used in diabetes mellitus

In the ID cohort, 91% of those with diagnosis of diabetes mellitus had at least one prescription of drugs for diabetes mellitus, and 66% of those with at least one prescription had a diagnosis (Table 1). The corresponding numbers in the gPop cohort were 88% and 67%.

For both types of diabetes mellitus, people with ID were more likely than those in the gPop sample to be prescribed intermediate or long-acting insulins combined with fast-acting insulins as well as sulfonylureas (Table 2, Additional file 3). They were, however, less likely to be prescribed fast-acting insulins and analogues for insulin-dependent diabetes mellitus. Also, they were less likely to be prescribed combinations of oral blood glucose lowering drugs, dipeptidyl peptidase-4, and exenatide/liraglutide for non-insulin-dependent diabetes mellitus. The pattern of prescription (ID vs gPop) of the investigated drugs were similar for men and women. Furthermore, there were no differences between women and men in neither cohort (Table 3).

**Table 2:** Prescription of drugs used for diabetes mellitus and hypertension among those with at least one such diagnosis in a group of people with intellectual disability (ID) and referents from the general population (gPop)

	All		
	gPop	ID	ID vs gPop
	*n* (%)	*n* (%)	RR (95% CI)
Insulin-dependent diabetes mellitus	*n* = 174	*n* = 182	
Insulins and analogues	147 (84)	155 (85)	1.01 (0.80–1.26)
Fast-acting	103 (59)	80 (44)	**0.74 (0.55–0.99)**
Intermediate-acting	71 (41)	57 (31)	0.77 (0.56–1.09)
Intermediate/long-acting comb. fast-acting	52 (30)	96 (53)	**1.77 (1.26–2.47)**
Long-acting	65 (37)	62 (34)	0.91 (0.64–1.29)
Blood glucose lowering drugs excl. Insulins	96 (55)	126 (69)	1.26 (0.96–1.64)
Biguanides	91 (52)	112 (62)	1.18 (0.89–1.55)
Sulfonylureas	27 (16)	55 (30)	**1.95 (1.23–3.09)**
Combinations	4 (2)	2 (1)	NC
α glucosidase inhibitors	3 (2)	4 (2)	NC
Thiazolidinediones	5 (3)	3 (2)	NC
Dipeptidyl peptidase-4 inhibitors	8 (5)	2 (1)	NC
Repaglinide or nateglinide	10 (6)	7 (4)	0.67 (0.26–1.76)
Exenatide or liraglutide	1 (1)	3 (2)	NC
Non-insulin-dependent diabetes	*n* = 546	*n* = 664	
Insulins and analogues	252 (46)	338 (51)	1.10 (0.94–1.30)
Fast-acting	125 (23)	136 (20)	0.90 (0.70–1.14)
Intermediate-acting	131 (24)	129 (19)	0.81 (0.64–1.03)
Intermediate/long-acting comb. fast-acting	114 (21)	212 (32)	**1.53 (1.22–1.92)**
Long-acting	84 (15)	100 (15)	0.98 (0.73–1.31)
Blood glucose lowering drugs excl. Insulins	423 (77)	528 (80)	1.03 (0.90–1.17)
Biguanides	398 (73)	473 (71)	0.98 (0.86–1.12)
Sulfonylureas	150 (27)	230 (35)	**1.26 (1.03–1.55)**
Combinations	20 (4)	11 (2)	**0.45 (0.22–0.94)**
α glucosidase inhibitors	6 (1)	8 (1)	1.10 (0.38–3.16)
Thiazolidinediones	23 (4)	21 (3)	0.75 (0.42–1.36)
Dipeptidyl peptidase-4 inhibitors	42 (8)	2 (3)	**0.43 (0.26–0.72)**
Repaglinide or nateglinide	34 (6)	36 (5)	0.87 (0.55–1.39)
Exenatide or liraglutide	15 (3)	7 (1)	**0.38 (0.16–0.94)**
Hypertension	*n* = 1487	*n* = 814	
Diuretics	641 (43)	482 (59)	**1.37 (1.22–1.55)**
Beta blocking agents	953 (64)	497 (61)	0.95 (0.86–1.06)
Calcium channel blockers	792 (53)	362 (44)	**0.84 (0.74–0.95)**
Agents acting on the renin-angiotensin system	1184 (80)	543 (67)	**0.84 (0.75–0.93)**
ACE inhibitors	863 (58)	463 (57)	0.98 (0.88–1.10)
Angiotensin II antagonists	614 (41)	145 (18)	**0.43 (0.36–0.52)**

Statistically significant results are marked in bold

*RR* relative risk, *CI* confidence interval, *ID* intellectual disability, *gPop* general population, *NC* not calculated due to too few observations

**Table 3:** Prescription of drugs used for diabetes mellitus and hypertension among those with at least one such diagnosis in a group of people with intellectual disability (ID) and referents from the general population (gPop), stratified by sex

		gPop	ID	ID vs gPop
		*n* (%)	*n* (%)	RR (95% CI)
Insulin-dependent diabetes mellitus				
Insulins and analogues	Women	57 (85)	65 (83)	0.98 (0.69–1.40)
	Men	90 (84)	90 (87)	1.03 (0.77–1.38)
	Men vs women	0.99 (0.71–1.38)	1.04 (0.76–1.43)	
Fast-acting	Women	42 (63)	30 (38)	**0.61 (0.38–0.98)**
	Men	61 (57)	50 (48)	0.84 (0.58–1.23)
	Men vs women	0.91 (0.61–1.35)	1.25 (0.80–1.97)	
Intermediate-acting	Women	24 (36)	21 (27)	0.75 (0.42–1.35)
	Men	47 (44)	36 (35)	0.79 (0.51–1.22)
	Men vs women	1.23 (0.75–2.01)	1.29 (0.75–2.20)	
Intermediate/long-acting comb. fast-acting	Women	22 (33)	42 (54)	1.64 (0.98–2.75)
	Men	30 (28)	54 (52)	**1.85 (1.19–2.89)**
	Men vs women	0.85 (0.49–1.48)	0.96 (0.64–1.44)	
Long-acting	Women	29 (43)	25 (32)	0.74 (0.43–1.26)
	Men	36 (34)	37 (36)	1.06 (0.69–1.67)
	Men vs women	0.78 (0.48–1.27)	1.11 (0.67–1.84)	
Blood glucose lowering drugs excl. Insulins	Women	40 (60)	52 (67)	1.12 (0.74–1.69)
	Men	56 (52)	74 (71)	1.36 (0.96–1.92)
	Men vs women	0.88 (0.58–1.32)	1.07 (0.75–1.52)	
Biguanides	Women	37 (55)	46 (59)	1.07 (0.69–1.65)
	Men	54 (50)	66 (63)	1.26 (0.88–1.80)
	Men vs women	0.91 (0.60–1.39)	1.08 (0.74–1.57)	
Sulfonylureas	Women	13 (19)	21 (27)	1.39 (0.70–2.77)
	Men	14 (13)	34 (33)	**2.50 (1.34–4.66)**
	Men vs women	0.67 (0.32–1.44)	1.21 (0.71–2.09)	
Combinations	Women	1 (1)	2 (3)	NC
	Men	3 (3)	0 (0)	NC
	Men vs women	NC	NC	
α glucosidase inhibitors	Women	2 (3)	3 (4)	NC
	Men	1 (1)	1 (1)	NC
	Men vs women	NC	NC	
Thiazolidinediones	Women	3 (4)	0 (0)	NC
	Men	2 (2)	3 (3)	NC
	Men vs women	NC	NC	
Dipeptidyl peptidase-4 inhibitors	Women	3 (4)	2 (3)	NC
	Men	5 (5)	0 (0)	NC
	Men vs women	NC	NC	
Repaglinide or nateglinide	Women	3 (4)	4 (5)	NC
	Men	7 (7)	3 (3)	NC
	Men vs women	NC	NC	
Exenatide or liraglutide	Women	0 (0)	1 (1)	NC
	Men	1 (1)	2 (2)	NC
	Men vs women	NC	NC	
Non-insulin-dependent diabetes				
Insulins and analogues	Women	94 (51)	161 (52)	1.02 (0.79–1.32)
	Men	158 (44)	177 (50)	1.14 (0.92–1.42)
	Men vs women	0.87 (0.67–1.12)	0.97 (0.78–1.20)	
Fast-acting	Women	53 (28)	61 (20)	**0.69 (0.48–1.00)**
	Men	72 (20)	75 (21)	1.06 (0.77–1.47)
	Men vs women	0.70 (0.49–1.00)	1.08 (0.77–1.52)	
Intermediate-acting	Women	48 (26)	68 (22)	0.85 (0.59–1.23)
	Men	83 (23)	61 (17)	0.75 (0.54–1.04)
	Men vs women	0.89 (0.63–1.28)	0.79 (0.56–1.12)	
Intermediate/long-acting comb. fast-acting	Women	44 (24)	103 (33)	1.40 (0.98–1.99)
	Men	70 (19)	109 (31)	**1.59 (1.18–2.14)**
	Men vs women	0.82 (0.56–1.20)	0.93 (0.71–1.22)	
Long-acting	Women	36 (19)	40 (13)	0.67 (0.42–1.04)
	Men	48 (13)	60 (17)	1.28 (0.87–1.86)
	Men vs women	0.69 (0.45–1.06)	1.32 (0.89–1.97)	
Blood glucose lowering drugs excl. Insulins	Women	138 (74)	246 (79)	1.07 (0.87–1.31)
	Men	285 (79)	282 (80)	1.01 (0.86–1.19)
	Men vs women	1.07 (0.87–1.31)	1.01 (0.85–1.20)	
Biguanides	Women	127 (68)	225 (72)	1.06 (0.85–1.32)
	Men	271 (75)	248 (70)	0.93 (0.79–1.11)
	Men vs women	1.10 (0.89–1.26)	0.97 (0.81–1.16)	
Sulfonylureas	Women	51 (27)	105 (34)	1.23 (0.88–1.72)
	Men	99 (28)	125 (35)	1.29 (0.99–1.68)
	Men vs women	1.00 (0.72–1.41)	1.05 (0.81–1.36)	
Combinations	Women	7 (4)	7 (2)	0.60 (0.21–1.71)
	Men	13 (4)	4 (1)	NC
	Men vs women	0.96 (0.38–2.41)	NC	
α glucosidase inhibitors	Women	4 (2)	7 (2)	NC
	Men	2 (1)	1 (0)	NC
	Men vs women	NC	NC	
Thiazolidinediones	Women	7 (4)	7 (2)	0.60 (0.21–1.71)
	Men	16 (4)	14 (4)	0.89 (0.44–1.83)
	Men vs women	1.18 (0.49–2.87)	1.76 (0.71–4.37)	
Dipeptidyl peptidase-4 inhibitors	Women	17 (9)	13 (4)	**0.46 (0.22–0.94)**
	Men	25 (7)	9 (3)	**0.36 (0.17–0.79)**
	Men vs women	0.76 (0.41–1.41)	0.61 (0.26–1.43)	
Repaglinide or nateglinide	Women	11 (6)	20 (6)	1.09 (0.52–2.27)
	Men	23 (6)	16 (5)	0.71 (0.38–1.34)
	Men vs women	1.08 (0.53–2.22)	0.71 (0.37–1.36)	
Exenatide or liraglutide	Women	3 (2)	5 (2)	NC
	Men	12 (3)	2 (1)	NC
	Men vs women	NC	NC	
Hypertension				
Diuretics	Women	307 (49)	220 (61)	**1.26 (1.06–1.50)**
	Men	334 (39)	262 (57)	**1.48 (1.26–1.74)**
	Men vs women	**0.80 (0.68–0.93)**	0.94 (0.78–1.12)	
Beta blocking agents	Women	415 (66)	216 (60)	0.91 (0.78–1.08)
	Men	538 (63)	281 (62)	0.98 (0.85–1.14)
	Men vs women	0.95 (0.84–1.08)	1.02 (0.86–1.22)	
Calcium channel blockers	Women	317 (50)	148 (41)	**0.82 (0.68–1.00)**
	Men	475 (55)	214 (47)	**0.85 (0.72–1.00)**
	Men vs women	1.10 (0.95–1.27)	1.14 (0.92–1.40)	
Agents acting on the renin-angiotensin system	Women	455 (72)	223 (62)	0.86 (0.73–1.01)
	Men	729 (85)	320 (70)	**0.83 (0.72–0.94)**
	Men vs women	**1.18 (1.05–1.32)**	1.13 (0.95–1.34)	
ACE inhibitors	Women	319 (51)	189 (53)	1.04 (0.87–1.25)
	Men	544 (63)	274 (60)	0.95 (0.82–1.10)
	Men vs women	**1.25 (1.09–1.44)**	1.14 (0.95–1.37)	
Angiotensin II antagonists	Women	259 (41)	60 (17)	**0.41 (0.31–0.54)**
	Men	355 (41)	85 (19)	**0.45 (0.36–0.57)**
	Men vs women	1.01 (0.86–1.18)	1.11 (0.80–1.55)	

Statistically significant results are marked in bold.

*RR* relative risk, *CI* confidence interval, *ID* intellectual disability, *gPop* general population, *NC* not calculated due to too few observations

Among those with insulin-dependent diabetes mellitus within the ID cohort, people in special housing were more likely to be prescribed fast- (RR 1.25 [0.73–2.14]), intermediate- (1.80 [0.89–3.68]), and long-acting (1.06 [0.59–1.90]) insulins and analogues for injection, as well as sulfonylureas (1.52 [0.77–3.02]). They were less likely to be prescribed insulins and analogues (0.94 [95% CI 0.66–1.34]), intermediate- or long-acting combined with fast-acting insulins and analogues for injection (0.78 [0.51–1.21]), blood glucose lowering drugs excluding insulins (0.95 [0.64–1.42], and biguanides (0.88 [0.58–1.34]). None of these results were, however, statistically significant. Other drugs were not investigated due to too low numbers.

Among those with non-insulin-dependent diabetes mellitus within the ID cohort, people in special housing were more likely to be prescribed intermediate-acting insulins and analogues for injection (RR 1.21 [0.81–1.82]) and sulfonylureas (1.11 [0.83–1.49]). They were less likely to have been prescribed insulins and analogues (0.98 [95% CI 0.68–1.07]), fast- (0.99 [0.68–1.44]) and long-acting (0.78 [0.51–1.18]) insulins and analogues for injection, intermediate- or long-acting combined with fast-acting insulins and analogues for injection (0.83 [0.62–1.11]), blood glucose lowering drugs excluding insulins (0.97 [0.80–1.17], and biguanides (0.92 [0.76–1.12]). None of these results were, however, statistically significant. Other drugs were not investigated due to too low numbers.

### Prescription of drugs for hypertension

In the ID cohort, 98% of those with diagnosis of hypertension had at least one prescription of drugs to treat hypertension, and 23% of those with at least one prescription had a diagnosis (Table 1). The corresponding numbers in the gPop cohort were 98% and 38%.

Among those with at least one diagnosis of hypertension, a higher percentage among people with ID than in the gPop sample were prescribed diuretics at least once during the study period (Table 2). The opposite, i.e. lower percentage of people with prescription among people with ID, was seen for calcium channel blockers and agents acting on the renin-angiotensin system. The latter was driven by a lower prescription of angiotensin II antagonists in the ID cohort. The patterns were similar when stratifying by sex. Within the gPop cohort, men were less likely than women to be prescribed diuretics, and more likely to be prescribed agents acting on the renin-angiotensin system, specifically ACE inhibitors (Table 3). No sex differences were found within the ID cohort. However, special housing was associated with statistically significantly lower risks of prescription of beta blocking agents (RR 0.75 [95% CI 0.67–0.84]), calcium channel blockers (0.70 [0.60–0.81]), agents acting on the renin-angiotensin system (0.67 [0.61–0.76]), ACE inhibitors (0.68 [0.60–0.77]), and angiotensin II antagonists (0.61 [0.49–0.75]), but not diuretics (1.04 [0.94–1.16]).

## Discussion

Women, but not men, with ID were more likely than the general population to have diabetes mellitus, regardless of proxy (diagnosis or prescription of drugs) for disorder. When determining presence of hypertension based on diagnosis, both men and women with ID were less likely to have hypertension than the general population. However, when considering prescription of drugs as a proxy for hypertension, the reduced prevalence was found only among men.

The major strength of the present study is the use of a national register for outcome assessments. In Sweden, all drugs prescribed for both diabetes mellitus and hypertension are sold by prescription only. Thus, no misclassification has been introduced by over-the-counter purchases.

We used the National Patient Register to identify people with a diagnosis of diabetes mellitus or hypertension. This register contains only diagnoses made in inpatient care and at outpatient specialist visits, not diagnoses made in primary care. In Sweden, the majority of those with insulin-dependent diabetes mellitus is treated at specialist centers, whereas most of the people with non-insulin-dependent diabetes mellitus are treated in primary care, with the exception of the more severe and complex cases [28]. In the present study, this is reflected in that only two thirds of those with prescription of drugs for diabetes mellitus had a recorded diagnosis of diabetes mellitus. That the percentage is similar in both the ID and the gPop cohort suggests that the failure to include those with diagnosis only in primary care have not biased the results when comparing the two cohorts. However, a similar percentage would also occur if people with ID have a higher prevalence of diabetes mellitus but are more likely to be undiagnosed and/or untreated, e.g. due to differences in access to primary care. This must be taken into consideration when interpreting the patterns of drug prescriptions in the different cohorts. Moreover, it must be acknowledged that the people included in the two cohorts may not be representative of all people with diabetes mellitus, and that the results might be generalizable only to people with severe or complex types of diabetes mellitus.

A potential weakness with the present study is the inability to adjust for potential confounders, as information on such were not available in any of the registers used. Although sex and age were taken into account in the matching of the referent cohort, we were not able to consider other factors that are known to be risk or protective factors for diabetes mellitus or hypertension, as well as differing in prevalence among people with ID and the general population. These include e.g. body mass index (BMI) and obesity [29–31], smoking [30, 32, 33], and alcohol use [30, 34, 35].

People in the ID cohort had a higher prevalence of diabetes mellitus (overall, i.e. not stratified by insulin-dependence), regardless of which proxy was used. This was driven by a high prevalence among the women with ID. Sex comparisons within each cohort and cohort comparisons within each sex revealed that women from the general population had a lower prevalence of diabetes mellitus than the other three groups. A different way to put this is that women with ID did not have the prevalence expected for women, but rather the same prevalence as found among the men. Women with ID are at greater risk of obesity and overweight than men with ID [36]. As a high BMI is one of the major risk factors for diabetes mellitus, a high prevalence of overweight among women with ID may be an explanation for their higher prevalence of non-insulin-dependent diabetes mellitus.

The prescription patterns were similar among those with insulin-dependent and non-insulin-dependent diabetes mellitus, in that people with ID were more likely to be prescribed combination drugs and sulfonylureas. Combination drugs are easier to handle than single drugs, both for the person with ID and for his or her carer. However, they also decrease the flexibility in adjusting the treatment based on individual needs. People with ID were also more likely to be prescribed sulfonylureas. For non-insulin dependent diabetes mellitus, the Swedish Medical Products Agency, recommend metformin, a biguanide, as first-line treatment [37]. Sulfonylurea, which is an older drug, should only be used when there is a contra-indication or intolerance for metformin. Our assessment of prescriptions is based on data for the entire study period, i.e. 2006–2012. Thus, our data would be in agreement with a pattern of changing from sulfonylurea to metformin during the study period. However, when looking at yearly prescriptions rather than prescriptions for the whole study period, there is no obvious trend over time (data not shown). Thus, the results for treatment of diabetes mellitus suggest that people with ID to a greater extent than the general population are prescribed drugs which are not recommended by the Medical Products Agency. At the same time, use of metformin is often limited for older people, due to comorbid conditions [20]. People with ID tend to have an earlier onset of the aging process [5, 38], and use of metformin may therefore be inappropriate at younger ages than in the general population.

When using only proxies for hypertension depending on diagnosis, people with ID had an almost halved risk of having hypertension as people in the general population, a result that was consistent for both men and women. This is opposite to what we expected, partly as previous studies have reported similar rates of hypertension among people with ID and the general population [8], and partly since people with ID display several risk factors associated with hypertension, such as congenital heart disease [39, 40] and obesity [36, 41]. When including also prescription of drugs to treat hypertension in the proxy for the disorder, the results were somewhat closer to what we had anticipated. The discrepancy between hypertension defined by prescription and hypertension defined by diagnosis is not surprising, as drugs used to treat hypertension also have other indications. For example, diuretics may be used to treat edema, and beta-blocking agents may be used to treat migraine. However, it is noteworthy that among people with ID, the fraction with diagnosis among those with prescription was lower than in the gPop cohort. This could either be due to a higher prescription rate among those without hypertension among people with ID, or to differences in which type of care (primary, inpatient, outpatient specialist) diagnoses of hypertension are made. If the cause is the latter, i.e. if people with ID are more likely than the general population to get their hypertension diagnosis in primary care than in other types of care, this could explain the lower prevalence of hypertension found among people with ID in the present study. However, another reason may be that people with ID are underdiagnosed with respect to hypertension. This could happen e.g. if either they themselves or their carers fail to understand the signs of hypertension or communicate them to the physician, and if the physician due to this fails to make a correct diagnosis. Underdiagnosis would also occur if routine blood pressure checks are not made on older people with ID to the same extent as in the general population.

Among those with at least one diagnosis of hypertension, people with ID were more likely to be prescribed diuretics and less likely to be prescribed several of the other drugs investigated. Another way of putting it, is that people with ID are more likely to be prescribed older drugs, whereas newer alternatives are more often used in the general population. There may be several reasons for this. People without ID probably have more knowledge about their disease and treatment options than people with ID. Thereby, they can make higher demands on their physician to try newer treatment regimes. They may also be more aware of possible adverse effects and alert their physician to them and thereby get a different drug. With respect to people with ID, it is not unlikely that a physician who have prescribed a drug that improves the health of the patient is unwilling to change the treatment simply because newer options are available. Although newer drugs are not by default better than older ones, especially in specific patient groups such as older people with ID, difficulties for people with ID in recognizing and communicating adverse effects warrant monitoring and regular drug reviews in this group.

## Conclusions

As expected, people with ID had a higher prevalence of diabetes mellitus than the general population. There were also differences in treatment regimes, in that people with ID more often were prescribed less flexible but more easy to use medication, and to a lesser extent newer types of medication. Quite unexpectedly, people with ID had a lower prevalence of hypertension than the general population, especially when considering only diagnoses and not drug prescriptions. If this is due to failure to diagnose hypertension among people with ID, or differences in types of health care used, is unknown. Treatment regimens for hypertension also differed among people with ID and the general populations such that people with ID more often were prescribed older types of medications. Therefore, it is of great importance to investigate if the people with ID and diabetes mellitus or hypertension are subjected to the same regular drug review that is recommended for older people in general in order to decrease the risk of CVD in this population.

As the present study was register based, we were only able to present data on diagnoses made and medications prescribed. Research in clinical settings is needed to obtain further knowledge about the treatment regimens used among older people with ID, and whether these are in accordance with their health problems. Also, to assess how older people with ID experience their disease and the treatment and health care they receive.

## Additional files

Additional file 1: Number of people with each type of diabetes mellitus and hypertension diagnosis in a cohort of people with intellectual disability (ID) and a random sample from the general population (gPop). Note! Each person may have more than one type of diagnosis. (DOCX 15 kb)

Additional file 2: Number of people with at least one prescription of each drug used in diabetes mellitus and drugs for hypertension, respectively, during 2006–2012 in a group of people with intellectual disability (ID, *n* = 7936) and a same-sized sample from the general population (gPop). (DOCX 15 kb)

Additional file 3: Prescription of drugs used for diabetes mellitus among those with at least one such diagnosis in a group of people with intellectual disability (ID) and referents from the general population (gPop). (DOCX 15 kb)

## Abbreviations

ACE: Angiotensin-Converting-Enzyme
ATC: Anatomical Therapeutic Chemical
CI: Confidence Interval
CVD: Cardiovascular disease
GLM: Generalized Linear Model
gPop: General Population
ICD: International Statistical Classification of Diseases and Related Health Problems
ID: Intellectual Disability
RR: Relative Risk
