# Supplementary material for: Prevalence and treatment of diabetes mellitus and hypertension among older adults with intellectual disability in comparison with the general population
Source: BMC Geriatr. 2017 Nov 23;17:272. doi: 10.1186/s12877-017-0658-2 (PMC5701367; doi:10.1186/s12877-017-0658-2)
Supplement: Supplementary file 1 — Number of people with each type of diabetes mellitus and hypertension diagnosis in a cohort of people with intellectual disability (ID) and a random sample from the general population (gPop). Note! Each person may have more than one type of diagnosis. (DOCX 15 kb) [file 12877_2017_658_MOESM1_ESM.docx]

|  | gPop | ID |
| --- | --- | --- |
|  | n (%) | n (%) |
| **Insulin-dependent diabetes mellitus** | 174 (2) | 182 (2) |
| ...with coma | 0 (0) | 4 (0) |
| ...with ketoacidosis | 3 (0) | 3 (0) |
| ...with renal complications | 6 (0) | 5 (0) |
| ...with ophthalmic complications | 61 (1) | 83 (1) |
| ...with neurological complications | 9 (0) | 8 (0) |
| ...with peripheral circulatory complications | 15 (0) | 12 (0) |
| ...with other specified complications | 12 (0) | 10 (0) |
| ...with multiple complications | 24 (0) | 25 (0) |
| ...with unspecified complications | 27 (0) | 17 (0) |
| ...without complications | 115 (1) | 99 (1) |
| **Non-insulin-dependent diabetes mellitus** | 546 (7) | 664 (8) |
| ...with coma | 6 (0) | 16 (0) |
| ...with ketoacidosis | 4 (0) | 9 (0) |
| ...with renal complications | 22 (0) | 16 (0) |
| ...with ophthalmic complications | 106 (1) | 143 (2) |
| ...with neurological complications | 18 (0) | 16 (0) |
| ...with peripheral circulatory complications | 26 (0) | 21 (0) |
| ...with other specified complications | 21 (0) | 41 (1) |
| ...with multiple complications | 54 (1) | 42 (1) |
| ...with unspecified complications | 78 (1) | 73 (1) |
| ...without complications | 473 (6) | 569 (7) |
| **Malnutrition-related diabetes mellitus** | 1 (0) | 2 (0) |
| ...with unspecified complications | 0 (0) | 1 (0) |
| ...without complications | 1 (0) | 1 (0) |
| **Other specified diabetes mellitus** | 5 (0) | 6 (0) |
| ...with ophthalmic complications | 1 (0) | 0 (0) |
| ...with multiple complications | 1 (0) | 0 (0) |
| ...with unspecified complications | 1 (0) | 1 (0) |
| ...without complications | 3 (0) | 5 (0) |
| **Unspecified diabetes mellitus** | 106 (1) | 118 (1) |
| ...with coma | 0 (0) | 2 (0) |
| ...with ketoacidosis | 0 (0) | 1 (0) |
| ...with ophthalmic complications | 23 (0) | 31 (0) |
| ...with peripheral circulatory complications | 4 (0) | 4 (0) |
| ...with multiple complications | 0 (0) | 1 (0) |
| ...with unspecified complications | 33 (0) | 18 (0) |
| ...without complications | 47 (1) | 69 (1) |
| Unspecified diabetes mellitus | 3 (0) | 0 (0) |
| **Essential (primary) hypertension** | 1467 (18) | 795 (10) |
| **Hypertensive heart disease** | 36 (0) | 19 (0) |
| ...with (congestive) heart failure | 11 (0) | 8 (0) |
| ...without (congestive) heart failure | 27 (0) | 11 (0) |
| **Hypertensive renal disease** | 20 (0) | 10 (0) |
| ...with renal failure | 18 (0) | 10 (0) |
| ...without renal failure | 3 (0) | 1 (0) |
| **Hypertensive heart and renal disease** | 2 (0) | 5 (0) |
| ...with (congestive) heart failure | 0 (0) | 3 (0) |
| ...with renal failure | 1 (0) | 0 (0) |
| ...unspecified | 1 (0) | 2 (0) |
| **Secondary hypertension** | 36 (0) | 19 (0) |
| Renovascular hypertension | 4 (0) | 3 (0) |
| Hypertension secondary to other renal disorders | 7 (0) | 7 (0) |
| Hypertension secondary to endocrine disorders | 2 (0) | 0 (0) |
| Other secondary hypertension | 0 (0) | 1 (0) |
| Secondary hypertension, unspecified | 26 (0) | 10 (0) |

Diagnoses with ICD-10 codes E10-E14 or I10-I15 not included in the table were not made during the study period.
