# Supplementary material for: Prevalence and treatment of diabetes mellitus and hypertension among older adults with intellectual disability in comparison with the general population
Source: BMC Geriatr. 2017 Nov 23;17:272. doi: 10.1186/s12877-017-0658-2 (PMC5701367; doi:10.1186/s12877-017-0658-2)
Supplement: Supplementary file 2 — Number of people with at least one prescription of each drug used in diabetes mellitus and drugs for hypertension, respectively, during 2006–2012 in a group of people with intellectual disability (ID, n = 7936) and a same-sized sample from the general population (gPop). (DOCX 15 kb) [file 12877_2017_658_MOESM2_ESM.docx]

|  | gPop | | ID | |
| --- | --- | --- | --- | --- |
|  | n (%) | | n (%) | |
| **Drugs used in diabetes mellitus** |  |  |  |  |
| A10AB01: Insulin (human), fast-acting | 10 (0) | | 2 (0) | |
| A10AB04: Insulin lispro, fast-acting | 39 (0) | | 22 (0) | |
| A10AB05: Insulin aspart, fast-acting | 135 (2) | | 156 (2) | |
| A10AB06: Insulin glulisine, fast-acting | 7 (0) | | 5 (0) | |
| A10AC01: Insulin (human), intermediate-acting | 180 (2) | | 182 (2) | |
| A10AD01: Insulin (human), intermediate/long-acting comb fast-acting | 3 (0) | | 8 (0) | |
| A10AD04: Insulin lispro, intermediate/long-acting comb fast-acting | 21 (0) | | 30 (0) | |
| A10AD05: Insulin aspart, intermediate/long-acting comb fast-acting | 123 (2) | | 251 (3) | |
| A10AE04: Insulin glargine, long-acting | 94 (1) | | 117 (1) | |
| A10AE05: Insulin detemir, long-acting | 30 (0) | | 27 (0) | |
| A10BA02: Metformin | 680 (9) | | 818 (10) | |
| A10BB01: Glibenclamide | 107 (1) | | 200 (3) | |
| A10BB07: Glipizide | 75 (1) | | 101 (1) | |
| A10BB12: Glimepiride | 54 (1) | | 57 (1) | |
| A10BD03: Metformin and rosiglitazone | 11 (0) | | 7 (0) | |
| A10BD07: Metformin and sitagliptin | 13 (0) | | 8 (0) | |
| A10BF01: Acarbose | 8 (0) | | 12 (0) | |
| A10BG02: Rosiglitazone | 14 (0) | | 29 (0) | |
| A10BG03: Pioglitazone | 19 (0) | | 12 (0) | |
| A10BH01: Sitagliptin | 57 (1) | | 35 (0) | |
| A10BH02: Vildagliptin | 2 (0) | | 0 (0) | |
| A10BH03: Saxagliptin | 5 (0) | | 1 (0) | |
| A10BX02: Repaglinide | 44 (1) | | 45 (1) | |
| A10BX03: Nateglinide | 1 (0) | | 4 (0) | |
| A10BX04: Exenatide | 4 (0) | | 4 (0) | |
| A10BX07: Liraglutide | 19 (0) | | 4 (0) | |
| **Drugs used for hypertension** |  |  |  |  |
| C03AA03: Hydrochlorothiazide | 240 (3) | | 108 (1) | |
| C03AB01: Bendroflumethiazide and potassium | 31 (0) | | 17 (0) | |
| C03BA08: Metolazone | 2 (0) | | 8 (0) | |
| C03CA01: Furosemide | 759 (10) | | 1722 (22) | |
| C03CA02: Bumetanide | 5 (0) | | 6 (0) | |
| C03CA04: Torasemide | 5 (0) | | 2 (0) | |
| C03DA01: Spironolactone | 221 (3) | | 359 (5) | |
| C03DA04: Eplerenone | 10 (0) | | 0 (0) | |
| C03DB01: Amiloride | 22 (0) | | 82 (1) | |
| C03EA01: Hydroclorothiazide and potassium-sparing agents | 348 (4) | | 266 (3) | |
| C07AA03: Pindolol | 14 (0) | | 8 (0) | |
| C07AA05: Propranolol | 187 (2) | | 125 (2) | |
| C07AA07: Sotalol | 58 (1) | | 18 (0) | |
| C07AB02: Metoprolol | 1290 (16) | | 865 (11) | |
| C07AB03: Atenolol | 579 (7) | | 471 (6) | |
| C07AB07: Bisoprolol | 330 (4) | | 164 (2) | |
| C07AG01: Labetalol | 4 (0) | | 3 (0) | |
| C07AG02: Carvedilol | 53 (1) | | 17 (0) | |
| C07FB02: Metoprolol and felodipine | 34 (0) | | 21 (0) | |
| C08CA01: Amlodipine | 872 (11) | | 447 (6) | |
| C08CA02: Felodipine | 669 (8) | | 419 (5) | |
| C08CA03: Isradipine | 11 (0) | | 10 (0) | |
| C08CA05: Nifedipine | 48 (1) | | 32 (0) | |
| C08CA06: Nimodipine | 2 (0) | | 0 (0) | |
| C08CA13: Lercanidipine | 33 (0) | | 5 (0) | |
| C08DA01: Verapamil | 45 (1) | | 25 (0) | |
| C08DB01: Diltiazem | 48 (1) | | 18 (0) | |
| C09AA01: Captopril | 11 (0) | | 13 (0) | |
| C09AA02: Enalapril | 1487 (19) | | 1052 (13) | |
| C09AA03: Lisinopril | 25 (0) | | 11 (0) | |
| C09AA05: Ramipril | 313 (4) | | 214 (3) | |
| C09AA06: Quinapril | 5 (0) | | 1 (0) | |
| C09AA08: Cilazapril | 10 (0) | | 5 (0) | |
| C09AA09: Fosinopril | 2 (0) | | 0 (0) | |
| C09BA02: Enalapril and diuretics | 280 (4) | | 119 (1) | |
| C09BA03: Lisinopril and diuretics | 10 (0) | | 3 (0) | |
| C09BA05: Ramipril and diuretics | 28 (0) | | 5 (0) | |
| C09BA06: Quinapril and diuretics | 2 (0) | | 3 (0) | |
| C09BA08: Cilazapril and diuretics | 8 (0) | | 2 (0) | |
| C09BB10: Trandolapril and verapamil | 1 (0) | | 2 (0) | |
| C09CA01: Losartan | 523 (7) | | 205 (3) | |
| C09CA02: Eprosartan | 11 (0) | | 1 (0) | |
| C09CA03: Valsartan | 71 (1) | | 16 (0) | |
| C09CA04: Irbesartan | 72 (1) | | 25 (0) | |
| C09CA06: Candesartan | 424 (5) | | 146 (2) | |
| C09CA07: Telmisartan | 16 (0) | | 3 (0) | |
| C09DA01: Losartan and diuretics | 262 (3) | | 46 (1) | |
| C09DA02: Eprosartan and diuretics | 4 (0) | | 3 (0) | |
| C09DA03: Valsartan and diuretics | 53 (1) | | 9 (0) | |
| C09DA04: Irbesartan and diuretics | 37 (0) | | 7 (0) | |
| C09DA06: Candesartan and diuretics | 161 (2) | | 27 (0) | |
| C09DA07: Telmisartan and diuretics | 11 (0) | | 2 (0) | |
| C09DB01: Valsartan and amlodipine | 10 (0) | | 0 (0) | |

Drugs for diabetes with ATC-code A10 and drugs for hypertension with ATC-code C03, C07-C09 not included were not prescribed during the study period.
