# Supplementary material for: Prevalence and treatment of diabetes mellitus and hypertension among older adults with intellectual disability in comparison with the general population
Source: BMC Geriatr. 2017 Nov 23;17:272. doi: 10.1186/s12877-017-0658-2 (PMC5701367; doi:10.1186/s12877-017-0658-2)
Supplement: Supplementary file 3 — Prescription of drugs used for diabetes mellitus among those with at least one such diagnosis in a group of people with intellectual disability (ID) and referents from the general population (gPop). (DOCX 15 kb) [file 12877_2017_658_MOESM3_ESM.docx]

|  | Both insulin- and non-insulin dependent | | | Insulin-dependent only | | | Non-insulin-dependent only | | |
| --- | --- | --- | --- | --- | --- | --- | --- | --- | --- |
|  | gPop (n=123) | ID (n=131) | ID vs gPop | gPop (n=423) | ID (n=533) | ID vs gPop | gPop (n=51) | ID (n=51) | ID vs gPop |
|  | n (%) | n (%) | RR (95% CI) | n (%) | n (%) | RR (95% CI) | n (%) | n (%) | RR (95% CI) |
| Insulins and analogues | 109 (89) | 116 (89) | 1.00 (0.77‑1.30) | 143 (34) | 222 (42) | **1.23 (1.00‑1.52)** | 38 (75) | 39 (76) | 1.03 (0.66‑1.60) |
| Fast-acting | 71 (58) | 60 (46) | 0.79 (0.56‑1.12) | 54 (13) | 76 (14) | 1.12 (0.79‑1.58) | 32 (63) | 20 (39) | 0.63 (0.36‑1.09) |
| Intermediate-acting | 54 (44) | 44 (34) | 0.77 (0.51‑1.14) | 77 (18) | 85 (16) | 0.88 (0.64‑1.19) | 17 (33) | 13 (25) | 0.77 (0.37‑1.57) |
| Intermediate/long-acting comb. fast-acting | 44 (36) | 76 (58) | **1.62 (1.12‑2.35)** | 70 (17) | 136 (26) | **1.54 (1.16‑2.06)** | 8 (16) | 20 (39) | **2.50 (1.10‑5.68)** |
| Long-acting | 44 (36) | 48 (37) | 1.02 (0.68‑1.54) | 40 (9) | 52 (10) | 1.03 (0.68‑1.56) | 21 (41) | 14 (27) | 0.67 (0.34‑1.31) |
| Blood glucose lowering drugs excl. insulins | 78 (63) | 99 (76) | 1.19 (0.89‑1.60) | 345 (82) | 429 (80) | 0.99 (0.86‑1.14) | 18 (35) | 27 (53) | 1.50 (0.83‑2.72) |
| Biguanides | 74 (60) | 86 (66) | 1.09 (0.80‑1.49) | 324 (77) | 387 (73) | 0.95 (0.82‑1.10) | 17 (33) | 26 (51) | 1.53 (0.83‑2.82) |
| Sulfonylureas | 20 (16) | 46 (35) | **2.16 (1.28‑3.65)** | 130 (31) | 184 (35) | 1.12 (0.90‑1.41) | 7 (14) | 9 (18) | 1.29 (0.48‑3.45) |
| Combinations | 3 (2) | 2 (2) | NC | 17 (4) | 9 (2) | **0.42 (0.19‑0.94)** | 1 (2) | 0 (0) | NC |
| α glucosidase inhibitors | 2 (2) | 4 (3) | NC | 4 (1) | 4 (1) | NC | 1 (2) | 0 (0) | NC |
| Thiazolidinediones | 5 (4) | 2 (2) | NC | 18 (4) | 19 (4) | 0.84 (0.44‑1.60) | 0 (0) | 1 (2) | NC |
| Dipeptidyl peptidase 4 (DPP-4) inhibitors | 6 (5) | 2 (2) | NC | 36 (9) | 20 (4) | **0.44 (0.26‑0.76)** | 2 (4) | 0 (0) | NC |
| Repaglinide or A10BX03: Nateglinide | 9 (7) | 6 (5) | 0.63 (0.22‑1.76) | 25 (6) | 30 (6) | 0.95 (0.56‑1.62) | 1 (2) | 1 (2) | NC |
| Exenatide or A10BX07: Liraglutide | 1 (1) | 3 (2) | NC | 14 (3) | 4 (1) | NC | 0 (0) | 0 (0) | NC |

RR = relative risk; CI = confidence interval; ID = intellectual disability; gPop = general population; NC = not calculated due to too few observations
